# Neurophysiological and behavioral measures of pain during neonatal hip examination

**DOI:** 10.1002/pne2.12006

**Published:** 2019-09-13

**Authors:** Miriam Pettersson, Emma Olsson, Andreas Ohlin, Mats Eriksson

**Affiliations:** ^1^ Department of Paediatrics Faculty of Medicine and Health Örebro University Örebro Sweden; ^2^ Faculty of Medicine and Health School of Medical Sciences Örebro University Örebro Sweden; ^3^ Faculty of Medicine and Health School of Health Sciences Örebro University Örebro Sweden

**Keywords:** neonatal hip examination, neonatal pain

## Abstract

**Introduction:**

The aim of this study was to test the hypothesis that neonatal hip examination causes pain in newborns. Pain assessment using instruments such as the Premature Infant Pain Profile‐Revised (PIPP‐R) scale is recommended, but recently physiological and neurophysiological measures, for example, near‐infrared spectroscopy (NIRS) and galvanic skin response (GSR), have been used as well.

**Methods:**

Heart auscultation and hip examination were performed, and the response of the newborn was registered by NIRS optodes, GSR electrodes, and a pulse oximeter probe attached to the infant. The face of the newborn was filmed. Heart auscultation was used as a nonpainful reference.

**Results:**

The pain scores for hip examination were higher than for the heart auscultation. Near‐infrared spectroscopy showed a significant higher increase from baseline in oxygenated hemoglobin (HbO_2_) on both sides of the cortex at hip examination compared with at heart auscultation (*P* = .011 and *P* = .017). Mean PIPP‐R scores for the hip examination compared with heart auscultation increased from 3.0 to 8.1 (*P* = .000). The GSR analyses of hip examination compared with heart auscultation showed a significant increase in area under small peaks during the hip examination (*P* = .016), however, not when measured in peaks per second (*P* = .104). Interrater reliability was calculated for the NIRS interpretations, with an intraclass correlation coefficient (ICC) range of 0.93‐1.0 (*P* = .000).

**Discussion:**

Pain in newborns can have negative consequences, and pain prevention and treatment are therefore important. We conclude that neonatal hip examinations are painful and that the pain should be treated, for example, with oral sweet solution. This is a change from present routines during neonatal hip examination and is hoped to lead to a change in national guidelines.

## Graphical abstract

Neonatal hip examination is painful and should therefore be treated with for example oral sweet solution.

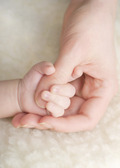

## 1 INTRODUCTION

Routine medical examinations of newborns are recommended by institutions such as the American Academy of Pediatrics^1^ and the British National Institute for Health and Care Excellence^2^ and are performed in most countries. In Sweden, as part of the healthcare system, all newborns are examined before discharge from the maternity ward to rule out innate abnormalities and ensure that the newborn is healthy.^3^ The examination that is performed by a physician usually takes about 5‐15 minutes and consists of 21 different components. Most of the components are quickly performed without agitating the newborn; however, some parts of the examination, such as the hip examination, appear to cause pain and discomfort.

Pain in newborns can have both short‐ and long‐term negative consequences. Previous studies have shown increased pain sensitivity and changes in future responsiveness of the neuroendocrine and immune systems in response to pain in early life.^4^, ^5^ Preventing and treating pain in newborns is therefore an important part of medical care.^6^ Healthy, full‐term newborns face several occasions of pain, such as the screening test for metabolic diseases, performed on all children in Sweden. Some of these painful procedures can be expected, and precautions should be made to minimize the pain.

There are several ways to recognize and assess pain in newborn infants. Pain assessment instruments such as the Premature Infant Pain Profile‐Revised (PIPP‐R)^7^, ^8^ are recommended and widely implemented, but recently some physiological and neurophysiological measures, such as near‐infrared spectroscopy (NIRS)^9^, ^10^, ^11^, ^12^ and galvanic skin response (GSR),^13^, ^14^ have been suggested to have pain assessment properties.

The PIPP‐R scale and its predecessor, PIPP, are two of the most extensively implemented pain scales and have been validated for assessing procedural pain in infants up to term age.^15^ The PIPP‐R consists of three behavioral indicators (facial expressions), two physiological indicators (changes in heart rate and oxygen saturation levels), and two contextual factors connected to infants’ ability to express pain (gestational age and alertness).^7^, ^8^, ^16^ A score >6 indicates pain. Near‐infrared spectroscopy is a spectroscopic method that provides information about the oxygen saturation of hemoglobin and can be used to measure, for example, cortical activation during a painful procedure.^9^, ^10^, ^11^, ^12^, ^17^ This technique uses light in the near‐infrared range (700‐1000 nm) that passes through tissue and bones but is absorbed by hemoglobin. The amount of light that is absorbed depends on the oxygen state of the tissue, where an increase in oxygenated hemoglobin (HbO_2_) reflects cerebral activation. Galvanic skin response measures skin conductance due to “emotional sweating” which reflects pain‐related activation of the sympathetic nervous system and has been used to measure neonatal pain in studies on term and preterm infants. Analyses of GSR are presented with a number of different variables, where area under small peaks and peaks per second has been shown to reflect pain in premature and term infants.^13^, ^14^, ^18^

Despite appearing clinically painful, the pain during newborn hip examinations has not been scientifically examined and newborns are not routinely given analgesia. Previous research on procedural pain in newborn infants has mainly focused on skin‐breaking procedures such as venipuncture, heel lancing, and injections.^19^ If pain can be objectively detected in newborns during these examinations, precautions can be taken and pain relieved, for example, by using sweet‐tasting solutions,^20^ thus lessening the risk for short‐ and long‐term negative consequences of pain. Through a previous study, made by our study group, the effect of using sweet‐tasting oral solution as pain relief during hip examination has been shown.^20^ After publication of that study, response has arisen, questioning whether hip examination indeed causes pain or not in the term newborn.

The aim of this observational study was therefore to test the hypothesis that neonatal hip examinations cause pain in newborns. The primary outcome was the change in oxygenated hemoglobin (HbO_2_) from before to after the procedure. Secondary outcomes were changes in GSR area under small peaks, changes in GSR peaks per second, and PIPP‐R score.

## 2 MATERIALS AND METHODS

Data collection for this study was conducted at the maternity ward at Örebro University Hospital from March to April 2018. The study was approved by the regional ethical review board (Dnr 2018/022). Parents of healthy, full‐term infants scheduled for a medical examination before discharge were approached and asked to participate in the study. Exclusion criteria were language (parents needed to speak enough Swedish to give informed consent), congenital malformations or other diseases in the infant, or any pain‐relieving drug administered to the newborn in the previous 24 hours.

**Figure 1:**
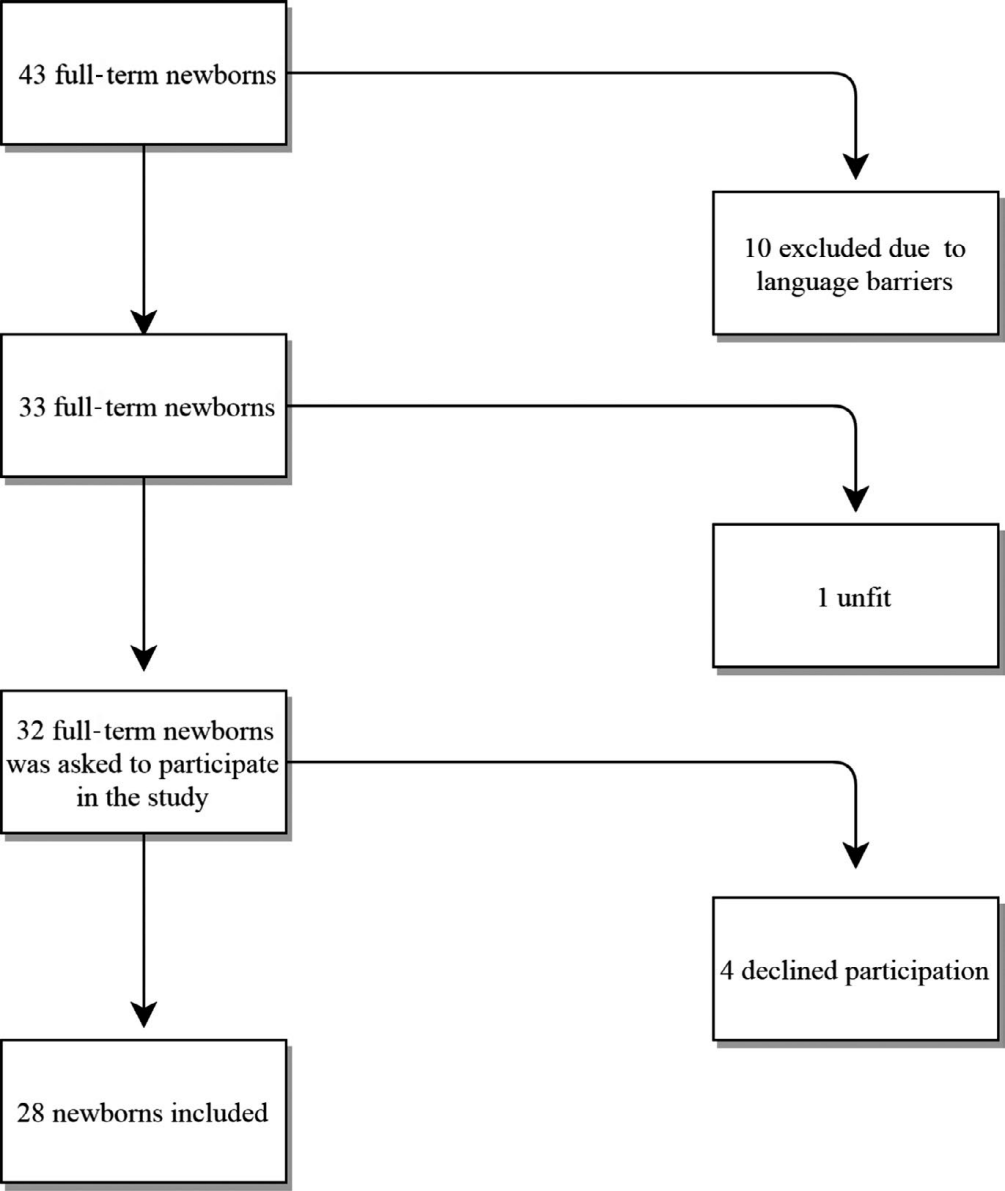
Flowchart showing the inclusion of infants in the study

Forty‐three infants were eligible but ten were excluded because of the language; four declined to participate, and one infant's parents were not approached because of psychiatric illness in the mother, which made her unfit to give valid consent to participation in the study. This left 28 healthy, full‐term infants who were included in the study after written consent from their parents (Figure 1).

Demographic and basic medical data were registered before the procedure started. The newborn infant was placed on a heated examination table, and a video camera was placed so that the newborn's face was filmed. A pulse oximeter probe (OxyTrend; Dräger Medical) was attached to one of the infant's feet and GSR electrodes (SensorMedics) to the sole of the other foot. Pulse oximetry values were filmed with a second video camera for later coding of PIPP‐R scores. Two NIRS optodes (NIRO 200NX; Hamamatsu Photonics) were attached bilaterally over the somatosensory cortex of the newborn. Heart auscultation, a procedure that does not cause pain in the newborn, was chosen to be used as a comparative to hip examination. This was done to be able to differentiate handling of the baby from hypothesized actual pain when examining NIRS, GSR, and PIPP‐R score. To synchronize all recordings, a vocal signal was recorded on the video simultaneously with pressing marker buttons on the NIRS and GSR equipment.

After obtaining stable signals on NIRS and GSR (the recordings were synchronized with each other and with the film recording), the heart auscultation was performed. After a minimum 30‐second waiting time for the readings to stabilize again, the hip examination consisting of the Barlow and Ortolani test^21^ for hip dysplasia was performed. The same neonatologist performed all examinations on all patients, and both procedures lasted for 10‐15 seconds each. Parents were invited to stay, but were asked not to touch their infant during the examination, and the infant was offered a finger or pacifier to suck on if they showed interest.

All recordings were continued 30 seconds after the procedure was finished and before the rest of the medical examination was conducted. After the examination, the film recording was used to perform the PIPP‐R coding. The captured bilateral NIRS measurements from channel 1 (right side of the head) and channel 2 (left side) were independently interpreted by two researchers (EO and MP), and the interrater reliability was calculated with intraclass correlation coefficient (ICC).

Outcome measure for NIRS was changed in oxygenated hemoglobin (HbO2)^9^ over a 30‐second period from the start of the procedure. The baseline value was determined from a stable 10 seconds prior to the start of the procedure. For GSR, we recorded area under small peaks and peaks per second during the first 30 seconds after start of the procedure.^13^, ^14^, ^18^ Also for PIPP‐R, the assessment time was the first 30 seconds from start of the procedure.^7^, ^8^, ^16^

Differences between the two studied components of the examination were analyzed with Wilcoxon signed‐rank test, and a *P*‐value of <.05 was considered significant, and we performed a Bonferroni‐Holm correction for multiple significance.

## 3 RESULTS

All included patients were healthy, full‐term newborns. Their demographic and basic background data are shown in Table 1. Because of technical difficulties, nine NIRS measurements, five GSR measurements, and three PIPP‐R measurements were excluded, leaving 17 (respectively 18, for channel 1 during heart examination) NIRS measurements, 23 GSR measurements, and 24 (respectively 25, for hip examination) PIPP‐R measurements to assess. The most common cause of technical difficulties was sliding of the NIRS optodes when the newborn moved during the examination, causing interference with the NIRS signals.

**Table 1:** Demographic data for the included infants

n = 28	n	Mean (SD)
Boys/girls	13/15	
Gestational age (wk)		39.9 (1.1)
Age at examination (h)		46.9 (45.1)
Birth weight (g)		3677.3 (521.1)
Apgar 1 min		9.0 (0.5)
Apgar 5 min		9.9 (0.4)
Apgar 10 min		9.9 (0.6)
Delivery mode (normal delivery/vacuum extraction/Caesarean section)	24/1/3	
Time since last feed (h)		0.8 (0.9)
Sucking during examination (yes/no)	8/20	
Parents present (Mother/father/both/unknown)	11/0/16/1	

**Figure 2:**
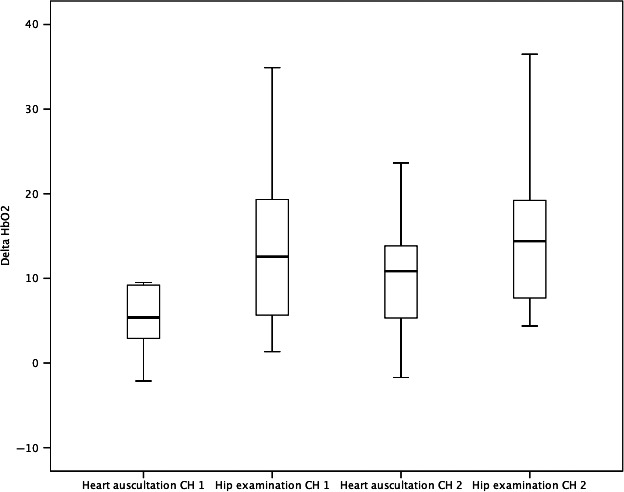
Boxplot diagram showing the changes in HbO2 during heart auscultation and hip examination, on both sides of the head. CH=NIRS channel

In summary, the results showed overall higher scores for the hip examination than for the heart auscultation, leading to the conclusion that hip examination is painful (Table 2). The main outcome, changes from baseline in HbO_2_ measured by NIRS, was significantly higher for hip examination than for heart auscultation on both sides of the cortex (*P* = .011 and *P* = .017) (Figure 2). The PIPP‐R mean value increased from 3.0 during the heart auscultation to 8.1 during the hip examination (*P* < .001). For GSR, the analyses of hip examination compared with heart auscultation showed a significant increase in the area under small peaks during the hip examination (*P* = .016), however, not when measured in peaks per second (*P* = .104). The Bonferroni‐Holm correction revealed no changes in significance.

**Table 2:** Pain measures during heart auscultation and hip examination. Values are median (q1‐q3)

	Heart auscultation	Hip examination	*P*‐value
NIRS, Delta HbO_2_ Channel 1	5.9 (3.1‐9.3)	12.6 (5.6‐19.4)	.020
NIRS, Delta HbO_2_ Channel 2	10.9 (5.3‐14.1	14.4 (7.7‐25.0)	.017
GSR, Small peaks	0.4 (0.2‐0.6)	0.6 (0.2‐2.2)	.016
GSR, Peaks per second	1.2 (1.1‐1.6)	1.2 (0.9‐1.4)	.104
PIPP‐R	1.5 (1.0‐3.0)	8.0 (6.0‐11.0)	<.001

The interrater reliability was calculated for the NIRS interpretations, with an ICC range of 0.93‐1.0 (*P* < .001).

## 4 DISCUSSION

Hip examination is performed in most countries during the routine newborn examination before discharge; observations showing that it causes pain should therefore be of interest to physicians worldwide. Whether neonatal hip examinations are painful or not has not been previously addressed in the scientific literature. As far as we know, this is the first scientific study to measure pain during neonatal hip examination. There are, as far as we know, no routines or guidelines available concerning treating potential pain during this procedure.

The fact that newborn infants can feel pain is no longer debatable, and compared with older children and adults, infants are more sensitive to pain; also, they are vulnerable to its long‐term negative effects,^22^ which is also true for full‐term infants.^23^ During the period before and after birth, the developing brain has a critical window of growth and development of the architecture of the brain. During this phase of increased plasticity, abnormal and repetitive exposure to pain can cause a certain amount of cell death.^22^ Despite this knowledge, infants are still subjected to painful procedures.^24^

Recognition and treatment of this pain to minimize the risk of adverse effects is imperative. Measuring pain in newborns is, however, a difficult task. Newborns cannot verbalize how or what they feel; instead, we are left to using different methods to assess pain. In this study, changes in HbO_2_, measured by NIRS, were the primary outcome and GSR and PIPP‐R were secondary outcomes. All three methods are well studied and have been used in previous research to assess pain in newborns. It is sometimes debated whether behavioral, physiological, or combined measures are the most valid and “true” sign of pain but there seems to be a consensus toward combined instruments.^25^ Neither NIRS nor GSR measures are specific to pain, but as we here show a significant increase in the response following hip examination compared with heart auscultation, further adds to the evidence of their usefulness as pain measures. In our study, a problem when using NIRS was sliding of the optodes due to head movements, which led to suboptimal measurements in some cases; these measurements were excluded. The difficulties of gaining stable NIRS measurements due to the baby moving are something to take into consideration in future studies when choosing measurement methods.

To be able to compare pain data with data from a nonpainful examination, we used heart auscultation as a reference. Our aim was to show that there is a difference between different components of the examination session and that hip examination causes not only discomfort but also pain.

To avoid bias in the interpretation of the results, we used two independent analyzers when analyzing the main outcome, NIRS HbO_2_ data. The ICC close to 1 indicates that the result is reliable.

The results of this study, using NIRS, PIPP‐R, and GSR, clearly indicate that neonatal hip examination is painful. Having established this, caregivers should focus on how to prevent and treat this pain.

There are different pain relief methods available for performing examinations and procedures on newborns, such as skin‐to‐skin contact, breastfeeding, or administering oral sweet solution. Depending on the character of the procedure, different methods are more, or less, suitable. Relieving pain through skin‐to‐skin contact or breastfeeding is possible during venipuncture, but is not as convenient during hip examination when the examiner needs a firm grip on the legs of the infant lying on a stable surface. Nonnutritive sucking can have a pain reliving effect; during the examination in our study, 8 of the newborns sucked on a finger or pacifier during both hip examination and heart auscultation. We have previously shown a pain‐relieving effect of oral glucose during hip examination. Repetitive doses of sucrose for longer periods to preterm infants have been suggested to have side effects,^26^ but administering oral sweet solution during a few hip examinations for full‐term infants therefore seems to be a valid option.^20^

We conclude that neonatal hip examinations are painful and that the pain should be treated, for example, with oral sweet solution. This is a considerable change from the present routines during neonatal hip examination; we recommend that national guidelines be changed accordingly.

## CONFLICTS OF INTEREST

The authors declare no conflicts of interest.

## **AUTHOR** **CONTRIBUTIONS**

M. Pettersson designed the study, conducted the inclusion of patients, and performed all examinations and analysation of the data, drafted the manuscript, and approved the final manuscript as submitted. E. Olsson assisted in designing the study, assisted in the measurements and analyzing of the data, critically reviewed and revised the manuscript, and approved the final manuscript as submitted. A. Ohlin assisted in designing the study and critically reviewed and revised the manuscript, and approved the final manuscript as submitted. M. Eriksson designed the study, assisted in measurements and analyzing the data, critically reviewed and revised the manuscript, and approved the final manuscript as submitted.
